# Idiopathic premature ventricular complexes treatment: Comparison of flecainide, propafenone, and sotalol

**DOI:** 10.1002/clc.24090

**Published:** 2023-08-02

**Authors:** Dejan Kojić, Anja Radunović, Zoran Bukumirić, Sasa Rajsic, Maša Sušić, Marija Marić, Vasko Žugić, Ružica Jurčević, Milosav Tomović

**Affiliations:** ^1^ Institute for Cardiovascular Diseases Dedinje Belgrade Serbia; ^2^ Institute of Medical Statistics and Informatics, Faculty of Medicine University of Belgrade Belgrade Serbia; ^3^ Department of Anesthesia and Intensive Care Medicine Medical University Innsbruck Innsbruck Austria

**Keywords:** AADs, antiarrhythmic drugs, flecainide, idiopathic ventricular arrhythmias, premature ventricular complexes, PVCs

## Abstract

**Background:**

Beta‐blockers (BB) or dihydropyridine calcium channel blockers (CCBs) are still the first choices in the treatment of idiopathic premature ventricular complexes (PVCs), with low‐modest efficacy. Antiarrhythmic drugs (AADs) of Ic class are moderate to highly efficient but the evidence on their benefits is still limited.

**Aim:**

To compare effectiveness and safety of flecainide, propafenone, and sotalol in the treatment of symptomatic idiopathic PVCs.

**Methods:**

Our single‐center retrospective study analyzed 104 consecutive patients with 130 medication episodes of frequent idiopathic PVCs treated with AADs flecainide, propafenone (Ic class) or sotalol (III class). The primary outcome was complete/near complete reduction of PVCs after medication episode (PVCs burden reduction >99%), and the secondary outcome was significant PVC burden reduction (≥80%).

**Results:**

The complete/near complete PVCs burden reduction occurred in 31% and was significant in 43% of treated patients. A reduction of PVC burden for >99% was achieved in 56% of patients on flecainide, in 11% of patients on propafenone (*p* = .002), and in 21% of patients receiving sotalol (*p* = .031). There was no difference between propafenone and sotalol (*p* = .174). A reduction of PVC burden for ≥80% was achieved in 64% of patients on flecainide, in 30% of patients on propafenone (*p* = .009), and 33% of patients on sotalol (*p* = .020). There was no difference between propafenone and sotalol (*p* = .661).

**Conclusions:**

The efficacy of AADs class Ic and III in the treatment of idiopathic PVCs was modest. Flecainide was the most effective AAD in the achievement of complete/near complete or significant PVC burden reduction, compared to propafenone and sotalol.

## Graphical abstract

The efficacy of antiarrhythmic drugs (AADs) class Ic and III in the treatment of idiopathic premature ventricular complexes (PVCs) was modest. Flecainide was the most effective AAD in the achievement of complete/near complete or significant PVC burden reduction, compared to propafenone and sotalol.

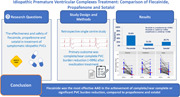

## 1 INTRODUCTION

Idiopathic ventricular arrhythmias are premature ventricular complexes (PVCs) or ventricular tachycardia (VT) in patients without structural heart disease. Treatment is indicated in case of symptomatic PVCs/VT and/or associated cardiac function deterioration.^1^

Catheter ablation (CA) of symptomatic idiopathic PVCs/VT is the first line and highly effective treatment option with low complications rate, particularly for the right ventricular outflow tract (RVOT) and fascicular types.^1^, ^2^, ^3^

Moreover, several drugs have been used in the treatment of idiopathic PVCs/VT, and current recommendations are based on a rather small or noncontrolled series. Although better than placebo, the use of beta‐blockers (BB) in the randomized controlled trials demonstrated clinically meaningful reduction of symptomatic outflow tract PVCs in only 12%–24% of patients.^4^, ^5^ The nondihydropyridine calcium channel blockers (CCBs) have demonstrated similar effectiveness in outflow tract PVCs, and are considered particularly useful for fascicular ventricular arrhythmias.^6^ Despite sparse evidence, BB or CCBs are considered to be the first choice for the treatment of symptomatic idiopathic PVCs from an origin other than the RVOT or the left fascicles.^2^ For patients who are unresponsive, or do not tolerate these drugs, and are not candidates for CA, treatment with antiarrhythmic drugs (AADs) such as flecainide, propafenone, sotalol, and amiodarone could be an alternative option.

The recent ESC Guidelines for the management of patients with ventricular arrhythmias recommends flecainide in such clinical scenarios. However, the evidence for this approach is scarce and there are no studies directly comparing the efficacy of these drugs.

In regard to amiodarone, considering its severe systemic toxicity, it is not indicated in the treatment of idiopathic PVCs and should be considered only in patients with left ventricular dysfunction.

The main aim of the present study was to compare the efficacy and safety of flecainide, propafenone, and sotalol in the treatment of idiopathic PVCs in a retrospective cohort of patients.

## 2 MATERIALS AND METHODS

### 2.1 Study aim and patient population

This is a single‐center, retrospective study aiming to compare the effectiveness and safety of propafenone, flecainide (AADs Class Ic), and sotalol (class III) in the treatment of symptomatic idiopathic PVCs. All consecutive symptomatic patients with frequent PVCs and preserved left ventricular systolic function referred to the Electrophysiology Outpatient Clinic of the Institute for Cardiovascular Diseases Dedinje were enrolled. The recruitment period was from 2019 to 2021.

The primary outcome was a complete or near‐complete reduction of PVCs after medication trial, defined as the proportion of patients with PVC burden reduction of >99% (cut‐off selected as it is approximately 99th percentile of a normal population).^7^ The secondary outcome was significant PVC burden reduction, defined as the proportion of patients with PVC burden reduction of ≥80%. The 80% cutoff was selected based on the earlier findings, where a reduction of ≥80% was sufficient to improve LVEF.^8^

We included all patients that were (1) older than 18 years, (2) having symptomatic frequent PVCs with a burden of ≥5% on 24‐h ECG Holter monitoring, and (3) LVEF of ≥ 50%. Exclusion criteria were (1) age ≤ 18 years, (2) previous myocardial infarction (identified by medical history or imaging), and (3) presence of structural heart disease in the cardiac imaging (moderate‐severe valvular heart disease, cardiomyopathy, scar, or wall motion abnormality). The decision to initiate therapy with a particular drug was based on the physician's preference.

### 2.2 Data acquisition

We obtained sociodemographic data of the patients; baseline clinical characteristics such as duration of arrhythmia, PVC burden before and after the treatment course, number of PVC morphologies, dominant QRS morphology (axis and bundle branch block pattern), etc. A medication trial was defined in days, as a time frame in which the patient was using one type of the prescribed antiarrhythmic drug, with a fixed dose. The minimum duration of the medication trial was 30 days. In cases when the dosage was increased, only the medication trial with the highest dosage was analyzed. The same patient could have been included in the multiple medication trials if the different AADs during separate periods of time were used. Combination with BB was possible according to the physician's decision. The efficacy of the medication in each trial was estimated with 24 h Holter ECG recordings, documented before and at least 30 days after the start of treatment.

Symptoms, 12‐lead ECG, echocardiographic findings, 24 h Holter ECG monitoring reports before and after treatment, potential reasons for drug discontinuation, or side effects were further obtained from the hospital information system. Transthoracic echocardiography was mandatory before treatment began (to assess LVEF, and to exclude structural heart disease) and after the medication episode.

An effect on symptoms after the medication trial was evaluated using a descriptive scale of symptomatic change: deterioration; no improvement; minimal improvement; moderate improvement and significant improvement. Safety and tolerability profiles of AADs were evaluated, including adverse drug reactions and drug discontinuation. Finally, the main clinical outcomes were all‐cause mortality, sudden cardiac deaths, and arrhythmia and/or heart failure‐related hospitalizations.

### 2.3 Statistical analysis

Depending on the type of variables and normality of the distribution, data were presented as frequency (percent), median (range) and mean ± standard deviation. For numeric data with non‐normal distribution and ordinal data, Mann–Whitney *U* test was used. The chi‐square test or Fisher's exact test was used to test the differences between nominal data (frequencies). We used logistic regression as the method for analyzing binary outcomes (Control PVC burden reduction >99% or PVC burden reduction ≥80%) and potential predictors. Independent variables that were significant (*p* < .05) in univariate models were used as the independent variables in the multivariate logistic regression model. Changes in the PVC burden before and after therapy were examined with a linear mixed effects modeling approach using R package lme4 version 1.1‐23. All *p*‐values less than .05 were considered as significant. We used the IBM SPSS Statistics 22 (SPSS Inc.) software package and the R software environment (R Core Team, 2021).

## 3 RESULTS

Over a period of three years, 104 patients were included in the study, Table 1. The mean age of included patients was 52.3 ± 16, and 59.2% (*n* = 77) were female. The median initial PVC burden was 15.0% (range 5.0%–48.0%), and the control median PVC burden after AADs therapy was significantly lower, 4.5% (range 0%–38.0%).

**Table 1:** Baseline demographic and clinical characteristics of included patients (*n* = 104).

Characteristics	All patients (*n* = 104)
Age	52.3 ± 15.8
Sex	
Female	77 (59.2%)
Male	53 (40.8%)
Comorbidities	
Hypertension	51 (39.0%)
Hyperlipidemia	22 (16.9%)
Diabetes mellitus	7 (5.9%)
COPD	3 (2.3%)
Duration of PVC in months	45 (5‐360)
PVC origin	71 (68.3%)
Non‐OT	10 (9.6%)
OT	61 (58.7%)
Not available	59 (56.7%)
Number of PVC morphology	**99** (**95.2%)**
1	68 (65.4%)
≥2	31 (29.8%)
Not available	31 (29.8%)
≥ 2 PVC morphologies, 1 dominant	**22** (**21.2%)**
Presenting symptoms	
No or minimal symptoms	40 (30.8%)
Palpitations	78 (60.0%)
Dyspnea	61 (46.9%)
Fatigue	18 (13.8%)
Dizziness/pre‐syncope	17 (13.3%)
Syncope	5 (3.9%)
Chest pain	21 (16.4%)
PVC burden before treatment (%)	15.0 (5.0–48.0)
PVC burden after treatment	4.5 (0.0–38.0)
LVEF (percentage), before treatment	57.2 ± 5.4
LVEF after treatment (%)	57.7 ± 5.8

*Note*: Data presented as mean ± standard deviation, median (range), or number of patients (%).

Abbreviations: COPD, chronic obstructive pulmonary disease; LVEF, left ventricular ejection fraction; OT, outflow tract; PVC, premature ventricular contraction; SD, standard deviation.

We identified 130 medication trials, with a maximum of three trials per patient, Table 2. Most of the patients had only one medication trial 81 (77.9%), 20 (19.2%) had two trials, while three patients had three trials of AADs. In total, AADs class Ic were most frequently prescribed—72 (55.4%) medication trials, while AAD class III (sotalol) was prescribed in 58 (44.6%) medication trials. The median duration of a specific AAD episode was 9 months (range 1–48). The duration of treatment was not significantly different between medication groups (*p* = .258; median and range duration: flecainid 10 (range 1–38) months, propafenone 13 (range 2–42) months, and sotalol 9 (range 2–48) months. The data about treatment trials are presented in Table 2.

**Table 2:** Treatment episodes (*n* = 130).

Characteristics	Medication episode (*n* = 130)
A trial of specific AAD class	
Class I	72 (55.4%)
Class III	58 (44.6%)
A trial of specific AAD	
Flecainide	45 (34.6%)
Propafenone	27 (20.8%)
Sotalol	58 (44.6%)
Number of trials of AAD	
One episode	81 (77.8%)
Two episodes	20 (19.2%)
Three episodes	3 (0.03%)
Combination with BB	39 (30.5%)
Duration in months of trial of specific AAD	9 (1–48)
Effect on symptoms	
Deterioration	5 (3.8%)
No improvement	50 (38.5%)
Minimal improvement	13 (10%)
Moderate improvement	31 (23.8%)
Significant improvement	31 (23.8%)
Reason for withdrawal/change of drug	
Unsatisfactory efficacy	33 (25.4%)
Side effect/intolerance of drug	1 (0.8%)
Spontaneous resolution	2 (1.5%)

*Note*: Data presented as median (range) or number of patients (%).

Abbreviations: AAD, antiarrhythmic drugs; BB, beta blocker.

Concerning the applied antiarrhythmic drugs, patients did not differ statistically in relation to age (*p* = .402), sex (*p* = .310) frequency of comorbidities (*p* = .548), or the duration of PVC (*p* = .402).

In our cohort of patients, there were 45 (34.6%) trials with flecainide. The most frequently prescribed daily dose was 200 mg/day in 53.3% (*n* = 24), 100 mg/day in 33.3% (*n* = 15), 50 mg/day in 8.9% (*n* = 4), and in two cases 300 mg/day 4.4% (Figure S1).

The median total daily dose was 200 mg [interquartile range [IQR]: 50.0–300.0], being 50% of maximal daily dose. Propafenone was prescribed in 27 (20.8%) medication episodes, with the most frequently prescribed daily dose of 450 mg/day in 44.4% (*n* = 12), 300 and 600 mg/day in 18.5% (*n* = 5), 150 and 750 mg/day in 7.4% (*n* = 2), and in one case 900 mg/day (3.7%). A median total daily dose of propafenone was 450 mg [IQR: 150.0–900.0], 50% of maximal daily dose. Finally, sotalol was used in 58 (44.6%) medication episodes, with most often prescribed daily dose of 160 mg/day in 68.9% (*n* = 40), 120 mg/day in 17.2% (*n* = 10), 80 mg/day in 6.9% (*n* = 4), 240 mg/day in 5.2% (*n* = 3), and in one case 200 mg/day (1.7%). The median total daily dose was 160 mg [IQR: 80.0–240.0], being 50% of maximal daily dose. The combination of beta‐blockers with AADs Ic has been reported in 39 (30.5%) medication trials. There was no difference in frequency of combination with BB between flecainide and propafenone (*p* = .257). In the multivariate logistic regression model, specific antiarrhythmic treatment was the only significant predictor of PVC burden reduction to <1% (flecainide vs. propafenone, *p* = .002 and flecainide vs. sotalol, *p* = .034). Comparing with flecainide, propafenone had almost 90% less probability for PVC burden reduction to <1%, and sotalol had almost 70% less probability (Table S1.).

In the multivariate logistic regression model, specific antiarrhythmic treatment was the only statistically significant predictor of PVC burden reduction of ≥80% (flecainide vs. propafenone, *p* = .011). Compared with flecainide, propafenone had 74% less probability for PVC burden reduction ≥80% (Table S2.).

During the follow‐up, a PVC burden reduction of >99%, was observed in 30.8% of treatment episodes, and a ≥ 80% burden reduction was reported in 43.1% of all 130 treatment episodes.

**Figure 1:**
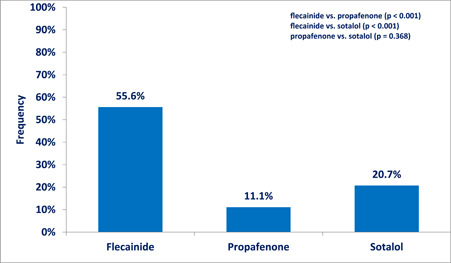
Proportion of treated patients with PVC burden reduction >99% after treatment. PVC, premature ventricular contraction.

Flecainide proved to be superior compared to propafenone and sotalol. In our study, 55.6% of patients treated with flecainide had complete/near complete resolution of PVCs, while the frequency was significantly lower after the use of sotalol (20.7%) or propafenone (11.1%) [flecainide versus propafenone, *p* < .001) and flecainide versus sotalol, *p* < .001)]. There was no difference in efficacy between propafenone and sotalol (*p* = .368), Figure 1.

**Figure 2:**
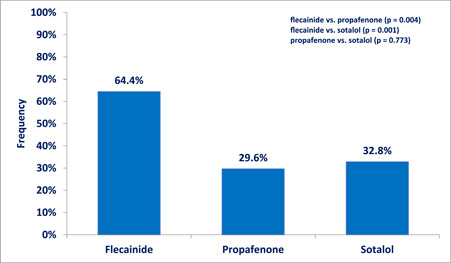
The proportion of treated patients with PVC burden reduction ≥ 80% after treatment. PVC, premature ventricular contraction.

PVC burden reduction of ≥80% was demonstrated in 64.4% of treated patients after the use of flecainide. The rate of significant PVC burden reduction was significantly lower after sotalol (32.8%, *p* = .001) or propafenone (29.6%, *p* = .004). We did not find any difference between propafenone and sotalol (*p* = .773), Figure 2.

**Figure 3:**
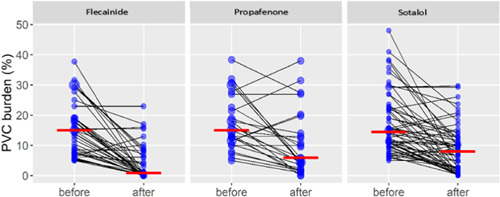
Dot‐plot comparison of flecainide, propafenone, and sotalol.

Moreover, the flecainide superiority compared to the propafenone or sotalol was confirmed with the dot‐plot presentation of the multivariate mixed effect model, Figure 3. Comparison of propafenone and sotalol didn't show a statistically significant difference in reduction of PVC burden (*p* = .661).

Individual values of PVC burden are presented with blue circles. The size of the circle depends on the number of respondents with the same value. The black lines show the direction toward the value at the last visit. Red dashes indicate medians. The dot‐plot of premature ventricular complexes burden before and after treatment is presented in Figure S2.

Regarding the effect on symptoms, 57.6% of patients had some symptomatic improvement. Of these, 23.8% had significant improvement, and the same proportion had moderate improvement, 10% had minimal improvement, and 3.8% had symptomatic deterioration. There was no change in symptoms in 38.5% (Figure S3).

The most frequent reasons for exclusion or change of the drug were unsatisfactory effect in 25.4% (*n* = 33), spontaneous resolution in 1.5% (*n* = 2), and side effects/intolerance of drug in 0.8% (*n* = 1). Adverse drug reactions were rare (9.2%, *n* = 12). Fatigue (6.2%) and dyspnea (1.5%) were the most commonly reported reactions. Visual disturbances (0.8%) and dizziness/syncope (0.8%) were rather rare. Significant QTc prolongation (>500ms) or significant QRSd increase (>120ms) were not observed, nor any severe adverse events, including proarrhythmic events, sudden cardiac death, or re‐hospitalizations.

## 4 DISCUSSION

In this retrospective study from a tertiary university center, we report on the efficacy and safety of three antiarrhythmic drugs used for treatment of idiopathic PVCs. We summarize that (1) all three analyzed drugs (flecainide, propafenone, and sotalol) are generally safe, (2) have modest effectiveness, and (3) we conclude that the flecainide has the highest rate of complete/near complete reduction of PVCs, being significantly more effective compared to propafenone or sotalol.

Our work brings the most recent evidence on the current management of frequent idiopathic PVCs in routine practice. To the best of our knowledge this is the first study directly comparing the effectiveness of three specific and most often prescribed AADs in the treatment of idiopathic PVCs. Moreover, different definitions of drug efficacy have been used, as the primary outcome of efficacy was more than 99% of PVC burden reduction in the analyzed population.

In our patient population, 30.8% of patients had a complete/near complete, and 43.1% had a significant PVC burden reduction. This data suggests that AAD treatment of idiopathic PVCs has limited efficacy, which is at best, modest. We found flecainide being the most effective AAD with PVC burden reduction of >99% in 56%, and PVC burden reduction ≥80% in 64% of treated patients. There was no statistical difference between propafenone and sotalol.

The previously reported efficacy of each drug class is inconsistent. The proportion of patients with relevant PVC reduction (≥75%–90%) ranged from 10% to 45% for BBs and 42% for AADs, being similar to our results.^9^

Beta blockers or nondihydropyridine CCBs (diltiazem or verapamil) were considered as a first‐line treatment for idiopathic PVCs. However, randomized clinical trials showed that BBs have rather small clinically relevant reduction in symptomatic outflow tract PVCs (12%–24%).^4^, ^5^ In a recent prospective study comparing the effectiveness and safety of BBs, non‐dihydropyridine CCBs, Class I and III antiarrhythmics to conservative therapy, AADs had only modest efficacy (56% of patients had significant reduction of PVC burden after treatment), being in line with our findings. However, the sample size of this study limited the ability to make conclusions about the relative effectiveness of individual class I and III drugs.^10^ In another study comparing relative efficacy of CA versus AADs in reduction of PVCs, CA showed better results (93%) than AADs (49%). Class I and III AADs were more effective (82%) than BB (36%, *p* < .001) or CCBs (43%, *p* < .001). It should be noted that the majority of patients in this study were treated with amiodarone, and only a few patients received propafenone, flecainide or sotalol.^11^ To close this gap, the present study directly compared those three AADs, identifying flecainide as the one with highest efficacy. These findings may help clinicians in the initial selection of the most appropriate antiarrhythmic drug for this specific group of patients.

Multivariate regression analysis did not identify any independent predictor of effectiveness, besides specific antiarrhythmic drug treatment. Even combination of BB with propafenone and flecainide did not show superior efficacy in comparation with individual antiarrhythmic drug treatment without BB.

In regard to the adverse drug reactions, we identified a rather low rate (9.2%), without special impact on the patient treatment. Fatigue was the most common complaint in our cohort, followed by dyspnea and dizziness/syncope, which is in line with the latest works.^5^, ^6^ None of the patients experienced any severe adverse events, death, or any proarrhythmic events of the analyzed antiarrhythmic drugs.

Finally, CA is the most effective treatment option for patients with symptomatic idiopathic PVCs, characterized with a low complication rate.^11^ Moreover, CA is recommended in the most recent European Society of Cardiology Guidelines for the management of patients with ventricular arrhythmias and prevention of sudden cardiac death, as the first line therapy for RVOT and fascicular type PVCs/VT. Although data are lacking, BBs or CCBs are still considered as the first choice for PVCs with an origin outside the RVOT or the left fascicles. Flecainide should be considered when CA is not available, not desired or has a high procedural risk.^2^ Our findings confirm the good safety profile and moderate efficacy of flecainide in the treatment of the idiopathic PVCs.

### 4.1 Further development and outlook

Despite the tremendous development of cardiology, many aspects of PVCs diagnosis and management are still unknown. The CA presents a rather invasive treatment option, that may lead to a resolution of PVCs but also to certain adverse events, which can be potentially severe. Moreover, in case of an unsuccessful CA, the only remaining therapy is pharmacological control of the arrhythmia.

The existing evidence on the PVC management in case the CA is not possible, or the patient is unresponsive to the first line drug treatment (BB or CCBs) is still sparse. The use of flecainide, propafenone, sotalol, or amiodarone can be considered, but the consensus on the best initial choice does not exist. The data on efficacy and safety of certain antiarrhythmic drug classes showed controversial results, missing to identify the best initial agent within the classes. With our work, we bring the proof of flecainide superiority when compared to propafenone or sotalol, accepting our main limitations—the single center and retrospective nature of the study. Moreover, our work brings new data pool of high methodological quality for possible future systematizations of evidence, as for example systematic reviews and meta‐analyses.

Further research should focus rather on the comparison of specific agents within the antiarrhythmic drug classes, including the intergroup comparisons. Future prospective studies with bigger samples are warranted. Until these data are available, our study showed moderate efficacy and acceptable safety of flecainide in the treatment of PVCs.

### 4.2 Limitations

Our study has several limitations. This is a single center retrospective study with its sample bias and potential confounding factors. The study design allowed multiple medication episodes per patient. The doses used in our study were relatively low, compared to previous clinical trials, especially for sotalol and propafenone. The decision to initiate therapy with a particular drug was based on the cautious assessment of the treating physician. However, a patient individualized approach respecting the most recent guidelines was applied, weighting the potential benefits of the treatment with possible risks. We used electronic medical documentation, and some data was not accessible for all patients. Moreover, the results of cardiac magnetic resonance were not available for the majority of patients. However, we included only patients with LVEF of ≥ 50% and we excluded patients with previous myocardial infarction, or any presence of structural heart disease in the cardiac imaging (moderate‐severe valvular heart disease, cardiomyopathy, scar, or wall motion abnormality). In almost half of the study patients, the origin of PVCs was undetermined. Hence, it was impossible to verify the effect of AADs on PVCs originating from specific source of origin. Moreover, we didn't use any specific quality of life questionnaire to estimate the patient's quality of life in regard to the reported outcomes.

Finally, for the PVC burden measurement, a 24 h ECG monitoring was used. This may lead to an underestimation or inaccuracy in identification of the PVC burden. However, the medical documentation of every patient was checked by two independent assessors, reducing the possible error to a minimum.

## 5 CONCLUSIONS

Propafenone, flecainide and sotalol are generally safe with only modest effectivity in the treatment of idiopathic PVCs. Flecainide is the most effective AAD in the achievement of complete/near complete or significant PVC burden reduction, compared to propafenone or sotalol. Further research should focus also on the comparison of specific agents within the antiarrhythmic drug classes, including intergroup comparisons.

## CONFLICTS OF INTEREST STATEMENT

The authors declare no conflict of interest.

## Supporting information

Supporting information.

## Data Availability

The data that support the findings of this study are available from the corresponding author upon reasonable request.
