# Supplementary material for: Idiopathic premature ventricular complexes treatment: Comparison of flecainide, propafenone, and sotalol
Source: Clin Cardiol. 2023 Aug 2;46(10):1220–6. doi: 10.1002/clc.24090 (PMC10577560; doi:10.1002/clc.24090)
Supplement: Supplementary file 1 — Supporting information. [file CLC-46-1220-s001.docx]

Idiopathic Premature Ventricular Complexes Treatment: Comparison of Flecainide, Propafenone and Sotalol

Supplementary material

Figure 1. Distribution of flecainide, propafenone and sotalol daily dose


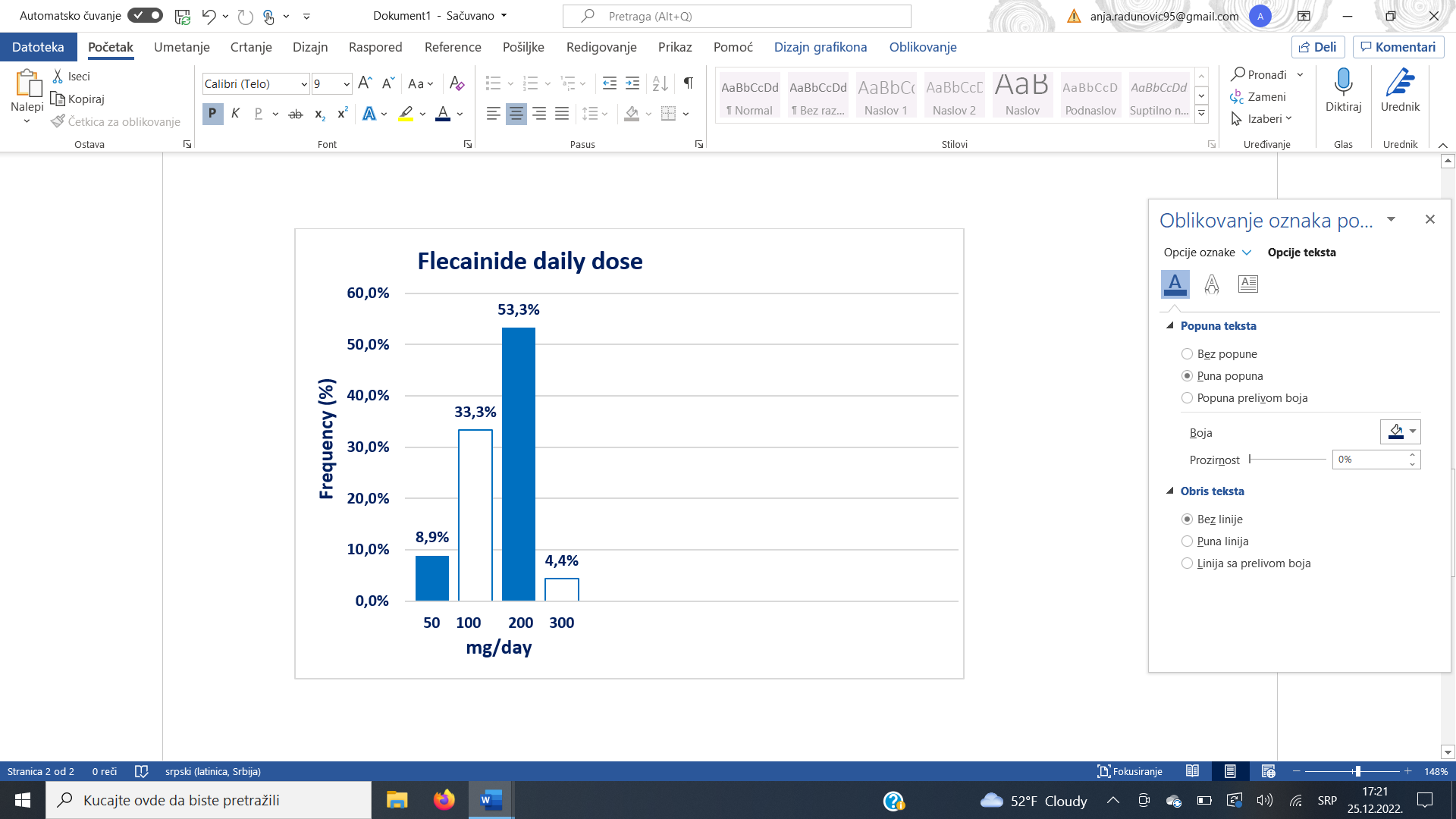

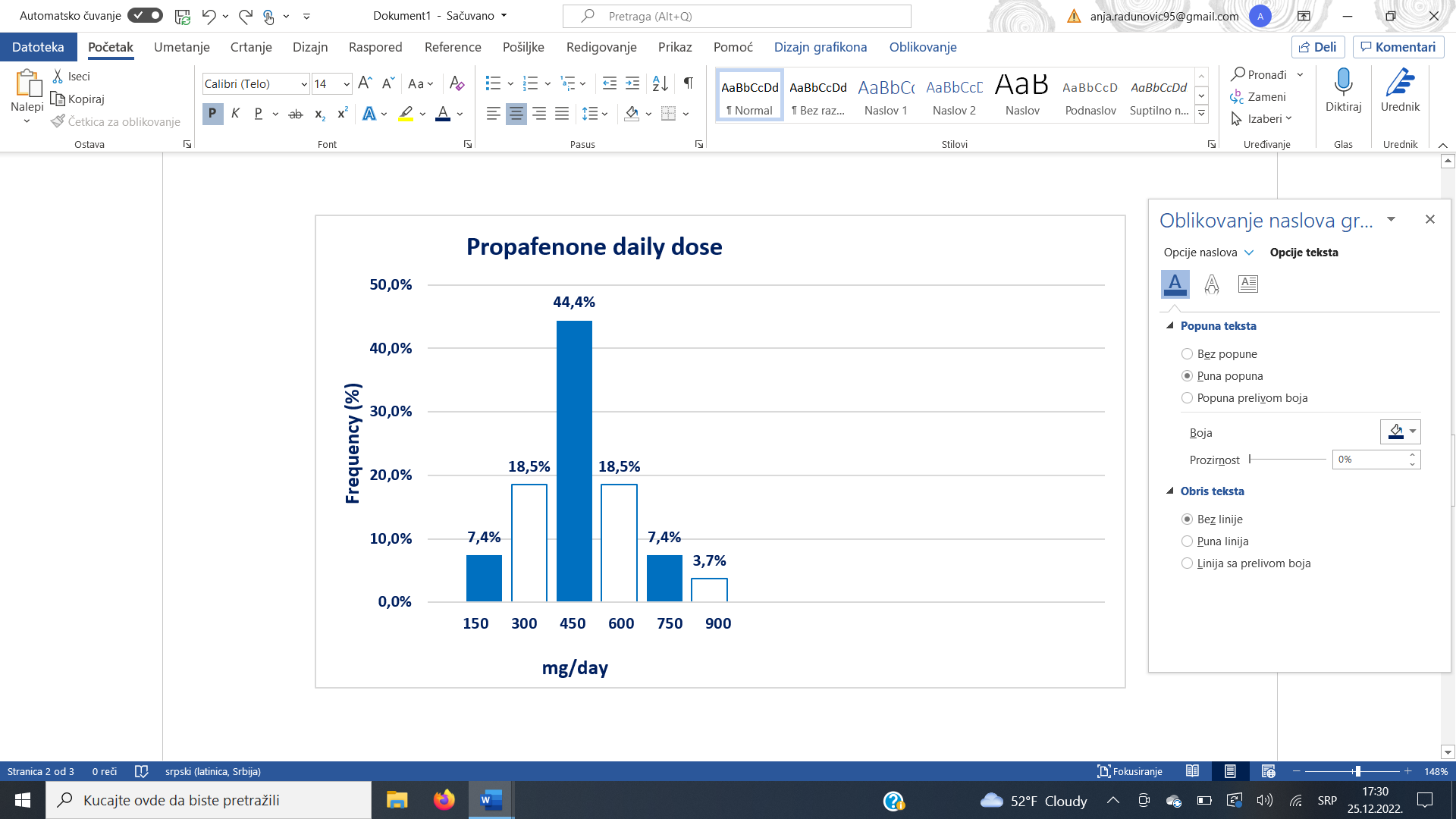

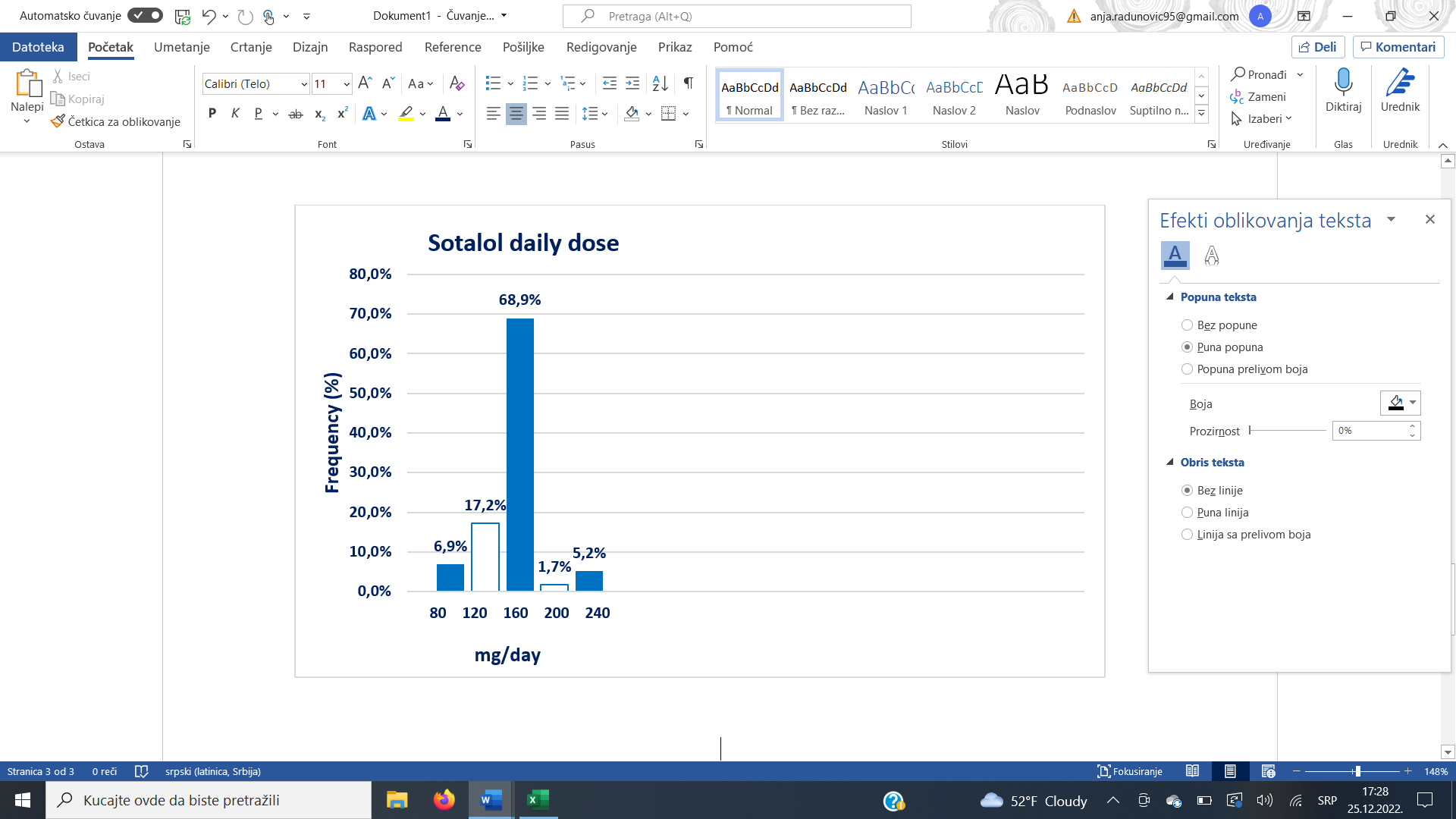


Table 1. Univariate and multivariate logistic regression analysis: Predictors of PVC burden reduction to <1%

| **Variable** | **Univariate analysis** | | **Multivariate analysis** | |
| --- | --- | --- | --- | --- |
|  | **OR 95% CI** | **p-value** | **OR (95% CI)** | **p-value** |
| Age (years) | 0.98 (0.96-1.01) | 0.104 |  |  |
| Sex (male) | 0.82 (0.38-1.76) | 0.613 |  |  |
| LVEF (%) | 1.04 (0.97-1.12) | 0.246 |  |  |
| PVC duration (months) | 1.00 (0.99-1.00) | 0.264 |  |  |
| PVC burden (%) | 0.95 (0.91-1.00) | 0.044 | 0.96 (0.91-1.00) | 0.069 |
| PVC morphology | 0.76 (0.17-3.32) | 0.716 |  |  |
| Non-responders | 1.77 (0.78-3.99) | 0.169 |  |  |
| Number of PVC morphologies | 0.92 (0.36-2.32) | 0.853 |  |  |
| Asymptomatic | 0.67 (0.29-1.54) | 0.344 |  |  |
| Drugs |  |  |  |  |
| Flecainide (reference category) | | |  |  |
| Propafenone | 0.10 (0.03-0.38) | 0.001 | 0.11 (0.03-0.44) | 0.002 |
| Sotalol | 0.21 (0.09-0.50) | <0.001 | 0.31 (0.10-0.91) | 0.034 |
| Combination with beta blockers | 2.61 (1.18-5.77) | 0.018 | 1.86 (0.61-5.64) | 0.275 |
| Duration of the medication trial | 0.98 (0.95-1.02) | 0.391 |  |  |

Abbreviations: OR: odds ratio, CI: confidence intervals, LVEF: left ventricular ejection fraction, PVC, premature ventricular contraction.

Table 2. Univariate and multivariate logistic regression analysis: Predictors of PVC burden reduction to ≥80%

| **Variable** | **Univariate analysis** | | **Multivariate analysis** | |
| --- | --- | --- | --- | --- |
|  | **OR 95% CI** | **p-value** | **OR (95% CI)** | **p-value** |
| Age | 0.99 (0.97-1.01) | 0.259 |  |  |
| Sex | 1.16 (0.57-2.36) | 0.674 |  |  |
| LVEF (%) | 1.04 (0.97-1.11) | 0.290 |  |  |
| PVC duration (months) | 1.01 (0.99-1.01) | 0.647 |  |  |
| PVC burden (%) | 1.01 (0.97-1.04) | 0.715 |  |  |
| Non-responders | 1.86 (0.85-4.07) | 0.120 |  |  |
| PVC morphology | 1.04 (0.27-4.08) | 0.953 |  |  |
| Number of PVC morphologies | 1.34 (0.57-3.15) | 0.503 |  |  |
| Without symptoms | 0.72 (0.33-1.54) | 0.393 |  |  |
| Drugs |  |  |  |  |
| Flecainide (reference category) | | |  |  |
| Propafenone | 0.23 (0.08-0.65) | 0.005 | 0.26 (0.09-0.73) | 0.011 |
| Sotalol | 0.27 (0.12-0.61) | 0.002 | 0.37 (0.14-1.001) | 0.0502 |
| Combination with beta blockers | 2.44 (1.13-5.27) | 0.023 | 1.64 (0.60-4.44) | 0.333 |
| Duration of medication trial | 1.03 (1.00-1.06) | 0.069 |  |  |

Abbreviations: OR: odds ratio, CI: confidence intervals, LVEF: left ventricular ejection fraction, PVC, premature ventricular contraction.

Figure 2**.** Dot-plot of premature ventricular complexes burden before and after treatment.


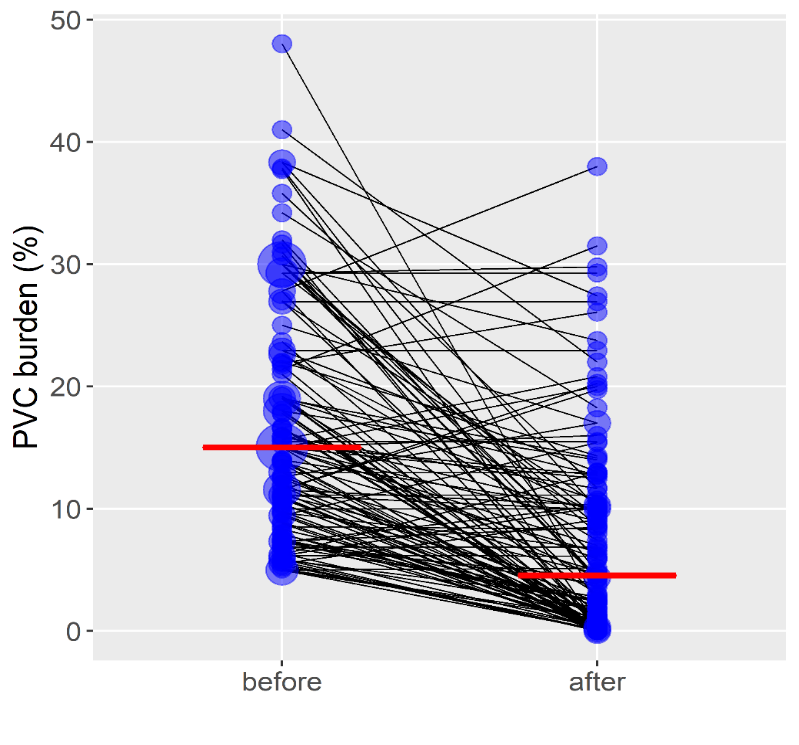


Figure 3. Effect of antiarrhythmic drug treatment on patient's symptoms.
